# Lying down with protective setae as an alternative antipredator defence in a non-webbing spider mite

**DOI:** 10.1186/2193-1801-2-637

**Published:** 2013-11-27

**Authors:** Shuichi Yano, Kanako Shirotsuka

**Affiliations:** Laboratory of Ecological Information, Graduate School of Agriculture, Kyoto University, Sakyo-ku, Kyoto, 606-8502 Japan

**Keywords:** Citrus red mite, Dorsal setae, Lying down, Predatory mite, Antipredator defence

## Abstract

An antipredator defence in the citrus red mite *Panonychus citri*, which does not produce protective webs, was examined experimentally. *P. citri* adult females lie down on citrus leaf surfaces with their dorsal setae (hair) directed in all upper directions. They seldom move in response to physical stimuli. Compared to normal lying females, both manipulated non-lying females and hair-removed females suffered higher predation by predatory mites. A predator approaching the body surface of a lying female inevitably created elasticity with a confronting seta, which eventually repelled the predator away from the female. These observations indicated that lying down with protective setae functions as an antipredator defence in *P. citri* females. This inflexible defence could also explain why the mite rarely runs away, even when it is consumed together with host plant leaves (via coincidental intraguild predation) by gigantic swallowtail caterpillars, against which protective setae are totally ineffective.

**Electronic supplementary material:**

The online version of this article (doi:10.1186/2193-1801-2-637) contains supplementary material, which is available to authorized users.

## Introduction

The life-dinner principle (Dawkins and Krebs 1979) predicts that antipredator behaviour should evolve faster than prey capture behaviour because successful defence means life to the prey, but only a lost meal to the predator. Spider mite and predatory mite systems offer one of the best examples of such predator–prey interactions: many spider mite species have developed co-operative defences against predators using three-dimensional protective webs (Saito 1986a; 1986b; Mori et al. 1999; Yano 2012), although the webs are ineffective against some predatory mites that specialize in preying on web-spinning spider mites (McMurtry et al. 1970; Sabelis and Bakker 1992; Shimoda et al. 2009). However, spider mites such as the citrus red mite [*Panonychus citri* (McGregor)] that do not produce protective webs (Saito 1983) also thrive under the same ecological conditions as mites producing protective webs. Since the two-dimensional webs produced by *P. citri* on leaf surfaces do not cover the body of the mite (Saito 1983), the webs obviously do not defend the mite from predators. Moreover, *P. citri* frequently moves between plant leaves (Wanibuchi and Saito 1983), indicating that the mite does not concentrate webs in a certain place on a plant. Therefore, we hypothesised that *P. citri* should have an alternative antipredator defence to producing protective webs.

Another unsolved mystery of *P. citri* is an apparently maladaptive trait: the mite rarely runs away even when it is dying because of coincidental intraguild predation from swallowtail caterpillars that consume mites together with citrus leaves (Shirotsuka and Yano 2012). In stable environments, traits that have a considerable negative impact on fitness are expected to be ultimately eliminated by natural selection. However, such traits can be maintained if they are advantageous in other circumstances (Nedelcu et al. 2011). Therefore, we hypothesised that by not moving in response to external stimuli, mites should realise a considerable benefit that overwhelms the cost of intraguild predation.

**Figure 1:**
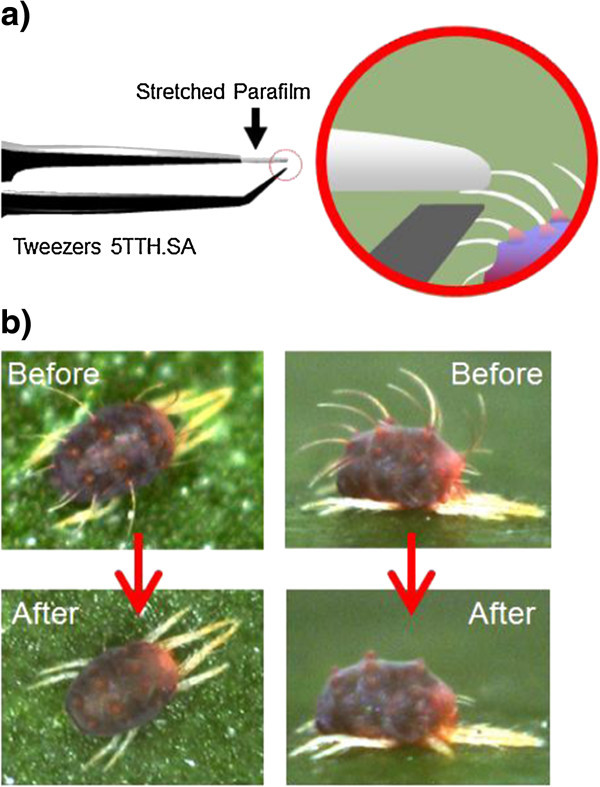
**Manipulation of hair-removed females. a)** Device used to pluck off individual setae. **b)** Appearance of *P. citri* adult females before (above) and after (below) hair removal.

The mite has a striking appearance (Figure 1b, above) for people other than acarologists: extremely long dorsal setae compared to the body size, a massive knot at the bottom of each seta, a perfect lying posture on a leaf surface and a vivid body colour like alpine flowers. In particular, the function of dorsal setae in mites has been poorly explored; they are only used as taxonomic characters. In this study, we experimentally removed dorsal setae from *P. citri* and directly demonstrated the defensive function of mite setae against predatory mites for the first time. We also determined the relationship between these traits and the antipredator strategy of the mite.

## Materials and methods

### Mites

The study population of *P. citri* was collected from a deciduous citrus shrub *Poncirus trifoliata* L. (Raf.) and separately maintained on leaf discs of kidney bean (*Phaseolus vulgaris* L.; bean population) or grapefruit (*Citrus paradise* Macf.; citrus population) that were pressed onto water-saturated cotton in Petri dishes (90 mm diameter, 14 mm depth). Although *P. citri* use citrus host plants in the wild, the mite is easily reared on bean leaf discs in the laboratory (Fukaya et al. 2013). The dishes were placed in transparent plastic containers and kept at 25 ± 2°C and 50 ± 5% relative humidity, with a photoperiod of 16-h L:8-h D (hereafter described as “laboratory conditions”). We maintained the population on each plant disc for more than three generations before conducting the following experiments.

For comparison, we also used a polyphagous spider mite *Tetranychus kanzawai* Kishida, which constructs complicated three-dimensional webs over leaf surfaces (Saito 1983). The study population of *T. kanzawai* was collected from a strawberry garden and maintained on bean leaf discs in the same manner described above.

The predatory mites *Euseius sojaensis* (Ehara) and *Neoseiulus womersleyi* Schicha are potential predators of *P. citri* (e.g. Osakabe et al. 1986; Inoue et al. 1987; Katayama et al. 2006). The former species is a typical generalist predator which feeds on both plant materials and spider mites, while the latter species mainly feeds on tetranychid mites (McMurtry and Croft 1997; Nguyen and Shih 2012). The study population of *E. sojaensis* was collected from kudzu vines *Pueraria lobata* (Willd.) Ohwi and was reared on tea pollen on bean leaf discs. The study population of *N. womersleyi* was collected from a hydrangea bush and reared on bean leaf discs that were heavily infested with *Tetranychus urticae* Koch as prey (30–50 female adults and individuals of other stages per leaf). The discs were maintained under laboratory conditions.

Adult female spider and predatory mites used in the following experiments were similar in size (≤0.5 mm). Detailed observations and transfers using a fine brush were only possible under a stereomicroscope. All tests described below were carried out between 1300 and 1700 h.

### Frequency of lying down females

To compare the frequency of lying down *P. citri* with that of *T. kanzawai* that produce protective webs, we observed the behavioural status (lying or otherwise) of the two species on bean leaf discs under laboratory conditions. We used bean leaves in order to equalize the structure of leaf surfaces on which the mites were observed. We introduced 10 newly emerged female adults of *P. citri* (bean population) onto each of seven bean leaf discs and *T. kanzawai* onto each of eight discs using a fine brush. The size of leaf discs was ca. 25 cm^2^. After 24 h of acclimation, we documented the behavioural status of the females, i.e. whether they were lying or acting otherwise (moving). Since we never observed *P. citri* females feeding while moving, they seemed to feed while lying down. We observed each female only once. The number of valid observations was 62 and 78 for *P. citri* and *T. kanzawai*, respectively. The proportions of lying females were compared using Fisher’s exact probability test.

### Response of lying females against a physical stimulus

**Figure 2:**
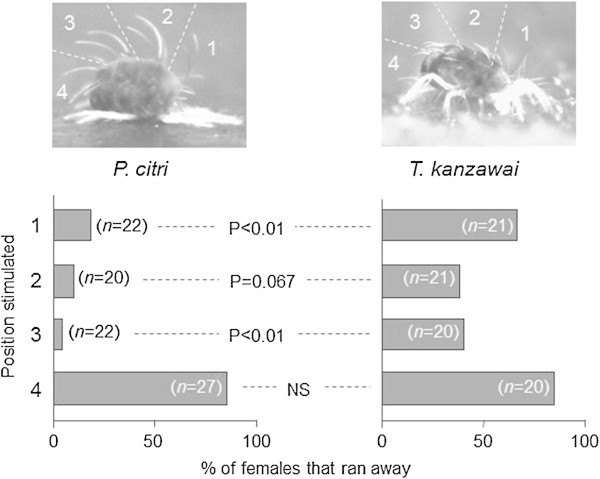
**Responses of lying females against physical stimuli.** Significantly fewer *P. citri* females ran away in response to stimuli on positions 1 or 3 compared to *T. kanzawai* females (Fisher’s exact probability test).

To compare the behavioural response of *P. citri* females against a physical stimulus with that of *T. kanzawai* females, we physically stimulated the dorsal setae of females that were lying on bean leaf discs after the above experiment. Since the major dorsal setae of *Panonychus* spider mites line in four rows (Ehara and Gotoh 1992), the dorsal positions of *P. citri*, including each row and corresponding position in *T. kanzawai*, were defined as positions 1–4 from the front (see Figure 2). Using the tip of a fine insect pin as a neutral substance, we stimulated the respective positions of lying *P. citri* and stationary *T. kanzawai* females and documented whether the females ran away or stayed motionless. In case when a target female had disturbed an adjacent untouched female, the latter female was excluded from the experiment. The number of valid observations for each combination of positions and mite species was >20. The proportions of females that ran away were compared using Fisher’s exact probability test.

### Function of lying down against predation

To examine the defensive function of the lying posture in *P. citri* females, comparing the predation rates between lying and non-lying females was necessary. However, preparing non-lying females against predators was impossible since untouched females usually lie down and rarely move in response to a stimulus (see Results and Additional file 1). Therefore, we created artificial non-lying females as follows. We used 2- to 4-day-old *P. citri* female adults from the citrus population. First, we fixed the female upside-down by gluing its dorsal setae with a droplet of glue (woodworking bond glue; Konishi Bond Adhesive Co., Tokyo, Japan) onto the surface of a 15 × 15-mm grapefruit leaf square that was pressed onto water-saturated cotton (upside-down females, *n* = 23). Second, we introduced a female that had been subjected to a sublethal intensity of ultraviolet irradiation (253.7 nm wavelength, 0.45 W m^–2^ for 1.5 h) using a GL-6 sterilisation lamp (6 W; Ultra-Violet Box; Sogo Rikagaku Glass Works Co., Kyoto, Japan) onto the leaf square (UV irradiated females, *n* = 23). Although the UV-irradiated females were still alive, they could not lie down like normal individuals. As a control, we introduced an untouched female onto the leaf square (control females, *n* = 24). We did not glue females in the normal lying posture as controls because it resulted in immediate death and depression of the female. Then, we introduced a 3- to 5-day-old starved female of either *E. sojaensis* or *N. womersleyi* that had been previously isolated for 48 h in a 1.5-ml microtube (Treff AG, Degersheim, Switzerland) with a water droplet. This was to promote immediate predation and to make it easier to judge predation because the transparent body of a starved predator turns a vivid vermillion after it consumes prey. After 24 h, we documented the proportion of consumed prey. The proportions were compared using Fisher’s exact probability test with Bonferroni step-down correction.

### Function of dorsal setae against predation

The best way to directly demonstrate the function of dorsal setae is to see what happens in their absence. However, cutting the dorsal setae off *P. citri* females without injuring them was practically impossible because of their small body size (≤0.5 mm). Instead, we plucked them off using the finest tweezers (5TTH.SA; Ideal-TeK SA, Balerna, Switzerland) as follows. We coated one tip of the tweezers with a fully stretched piece of Parafilm M (American National Can Co., Chicago, IL, USA). Then we plucked the setae off 2-day-old *P. citri* females because it was only possible to pluck setae on the second day after maturation (Yano personal observation). As we pinched individual seta using the tweezers under a stereomicroscope, the sticky coated tip trapped the target seta (Figure 1a). Hair removal from *P. citri* was possible due to the following factors. First, since *P. citri* females rarely run away in response to external stimuli, we could manipulate them while alive on leaf surfaces. Second, since each seta extends in an independent direction, we could pinch and remove them one by one, which was necessary because a seta could only be plucked along its direction. Finally, we found that the plucking of setae was only possible on the second day after female maturation. In addition, since seta removal required elaborate work under a powerful stereomicroscope, one must be careful to provide enough illumination to avoid weakening one’s eyesight (S. Yano personal experience).

We confirmed that all hair-removed 2-day-old females (*n* = 11) survived the following 24 h by maintaining them on 15 × 15-mm grapefruit leaf squares; the females lay down on the leaves as did untouched (hairy) individuals. We also confirmed that the number (± SE) of eggs oviposited in 24 h by hair-removed females (3.82 ± 0.48, *n* = 11) and untouched females (3.58 ± 0.29, *n* = 12) were equivalent (*P* = 0.80, Mann–Whitney *U*-test), suggesting that the both females had similarly fed and oviposited on the leaves. We confined hair-removed (*n* = 14) or hairy (control, *n* = 15) 2-day-old *P. citri* females on leaf squares. Then we introduced a starved *E. sojaensis* female onto each leaf square in the manner that was described above. After 24 h, we documented the proportion of consumed prey. The proportions were compared using Fisher’s exact probability test. After the experiment was concluded, we video-recorded a starved *E. sojaensis* attacking a hairy *P. citri* female on a leaf square to document the moment of an unsuccessful attack.

### The preferences of *P. citri* and predatory mites for upper and lower leaf sides

To compare the leaf-side preferences of *P. citri* with those of *E. sojaensis* and *N. womersleyi*, a detached grapefruit leaf was inserted into a 2-ml tube bottle filled with water, and the bottle was fixed so that the leaf sides were held horizontally. Water could not leak from the tube because of a small bottle neck. Then we introduced a female of *P. citri* or predatory mites onto the upper leaf-side and kept the setup under laboratory conditions. After 1 h, we recorded the leaf side on which the female had settled. The proportions of mites settled on upper and lower sides were compared using Fisher’s exact probability test with Bonferroni step-down correction.

## Results

### Frequency of lying down females

**Figure 3:**
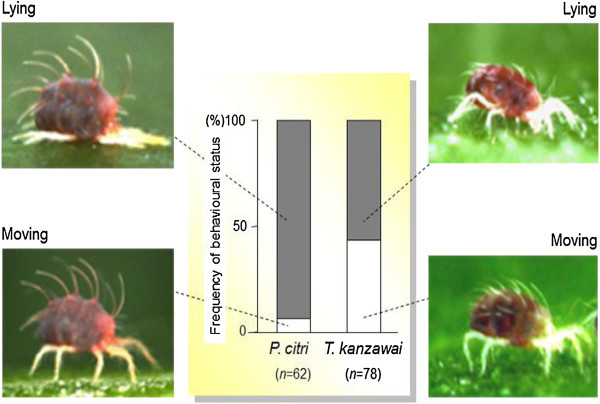
**Frequency of lying down females in** ***P. citri*** **and** ***T. kanzawai*** **.** The frequency of behavioural status differed significantly between the species (*P* < 0.0001, Fisher’s exact probability test).

The observed frequency of the lying posture was significantly higher in *P. citri* (93.5%) than *T. kanzawai* (56.4%), suggesting that *P. citri* allocated much more time to lying compared to *T. kanzawai* (Figure 3).

### Response of lying females against a physical stimulus

Significantly fewer *P. citri* females ran away in response to a physical stimulus on positions 1 or 3 compared to *T. kanzawai* females (Fisher’s exact probability test; Figure 2). The difference was nearly significant for position 2 (Figure 2). For these positions, *P. citri* females did not seem to move in response to physical stimuli as a rule. However, both *P. citri* and *T. kanzawai* females quickly ran forward in response to a stimulus on position 4 (Figure 2). Moreover, one can clearly see that setae forms and lying postures were different between the species: long setae of *P. citri* extend in independent directions, whereas short setae of *T. kanzawai* point in the same direction, and gaps between the mite body and the leaf surface were minimal in *P. citri* but variable in *T. kanazawai* (Figure 2).

### Function of lying down against predation

**Figure 4:**
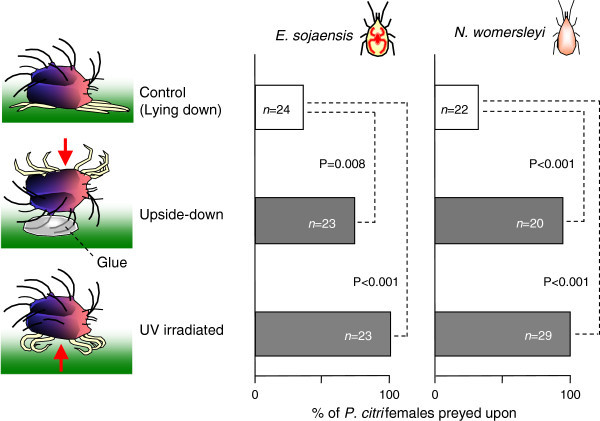
**Function of lying down against predation.** Significantly more *P. citri* females that could not lie down were consumed than normal ones (Fisher’s exact probability test with Bonferroni step-down correction). Arrows indicate directions from which predators could access disabled females.

Predatory mites could easily access both upside-down and UV-irradiated females from the females’ exposed underside where dorsal setae were absent. Significantly more *P. citri* females that were fixed upside-down were consumed compared to normal ones that could lay down (*P* = 0.0084 for *E. sojaensis* and *P* < 0.001 for *N. womersleyi*, Fisher’s exact probability test with Bonferroni step-down correction; Figure 4). Similarly, significantly more UV-C-irradiated *P. citri* females were consumed than normal ones (*P* < 0.001 for both predatory mites; Figure 4). The higher predation rates on *P. citri* females that could not lie indicate that lying down prevented predation.

### Function of dorsal setae against predation

**Figure 5:**
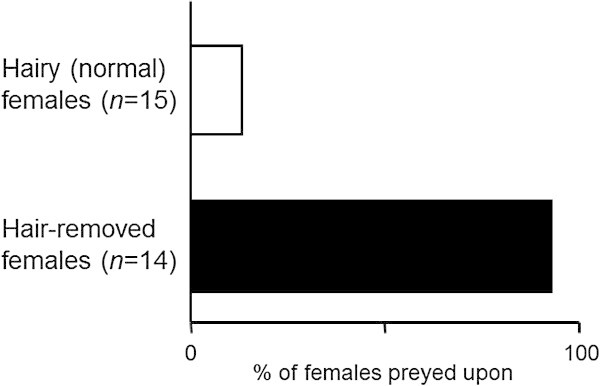
**Function of dorsal setae against predation.** Significantly more hair-removed females were killed than normal (hairy) ones in the presence of a predator (*P* < 0.0001, Fisher’s exact probability test).

Although both hair-removed and hairy (normal) *P. citri* females lay down, significantly more hair-removed females were killed than hairy ones in the presence of a predator (*P* < 0.0001, Fisher’s exact probability test; Figure 5), indicating that dorsal setae prevented predation.

The video recordings suggested that the setae of *P. citri* females prevent the approach of and attacks by *E. sojaensis* females (Additional file 1). A female *E. sojaensis* attempting to approach the body surface of a lying female was sometimes repelled by an elastic seta (Additional file 2).

### The preferences of *P. citri* and predatory mites for upper and lower leaf sides

**Figure 6:**
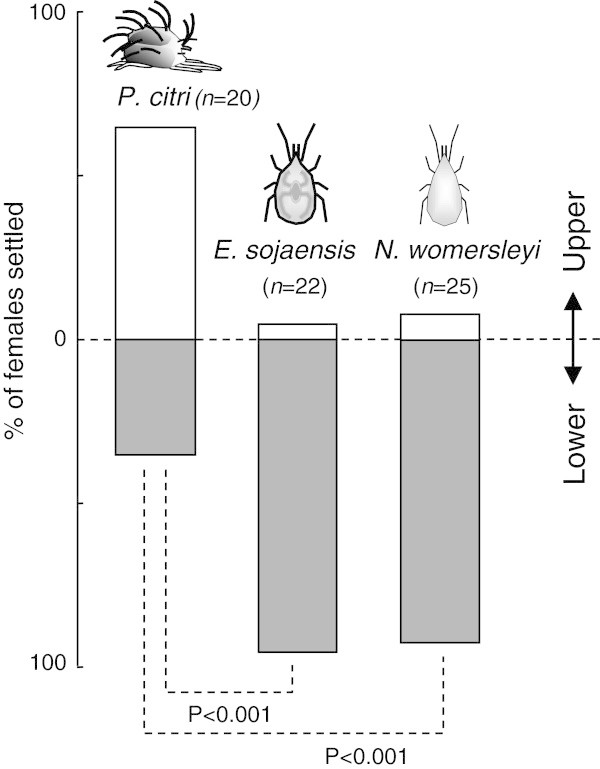
**Leaf-side preferences of** ***P. citri*** **and predatory mites.** Significantly more *P. citri* females settled on upper leaf-sides compared to predatory mite females (*P* < 0.001, Fisher’s exact probability test with Bonferroni step-down correction).

More than half of the *P. citri* females (65%) that were introduced onto the upper leaf-side remained on the side. In contrast, only one of 22 *E. sojaensis* females (4.5%) and two of 25 *N. womersleyi* females (8.0%) that were introduced onto the upper leaf-side remained on the side (*P* < 0.001, Fisher’s exact probability test with Bonferroni step-down correction, Figure 6), suggesting that predatory mites did not settle on an upper leaf-side as a rule.

## Discussion

The high frequency with which *P. citri* females lay on leaves suggested that this trait is a constitutive one. Lying *P. citri* females did not move in response to external physical stimuli as a rule, except when they were stimulated at the tail end (position 4). These *P. citri* females, and most *T. kanzawai*, females did run away against stimuli, suggesting that *P. citri* females that did not move in response to a stimulus should have a selective advantage.

From this viewpoint, we examined the defensive function of the lying posture of *P. citri* females by manipulating non-lying females. The higher consumption rate of non-lying females by the both predatory mites in turn suggested that the lying posture of *P. citri* females is effective against wide range of predatory mites. We also examined the defensive function of the dorsal setae of *P. citri* females by manipulating hair-removed females. Since hair-removed *P. citri* females readily survived in the absence of predators, the higher predation rate for hair-removed females demonstrated that dorsal setae functioned to prevent predation.

To consume prey spider mites, the mouthpart of the predatory mite should reach the prey body surface. However, massive knots at the bottom of each seta seemed to maintain the direction of the seta, which prevented predators from approaching the prey body surface. Since the setae of lying *P. citri* extend in all upper directions, regardless of the direction of attack, predators cannot avoid confronting a seta. As a result, predator’s efforts to approach the prey body surface inevitably create strong elasticity with the confronting seta, and the predator is physically repelled due to its own action (Additional file 2).

Since *P. citri* uses both the upper and lower leaf sides (Jones and Parrela 1984), the importance of repelling predators may depend on which leaf side the mite inhabits. The antipredator strategy will be highly effective on a lower leaf-side where the probability that a repelled predatory mite would come back to attack the same *P. citri* individual is negligible. In contrast, a predator that is repelled away on an upper leaf-side is more likely to remain on the side and may return to the same *P. citri* individual. However, we confirmed that the both predatory mites did not inhabit the upper leaf-side as a rule. Indeed, Sudo and Osakabe (2011) reported that 98% of predatory mites in the wild are found on a lower leaf-side. These results suggest that upper leaf-sides function as enemy-free spaces for *P. citri*, where its antipredator defence by lying down would be less effective. Fukaya et al. (2013) suggested that the cost to *P. citri* of being exposed to UV irradiation on the upper leaf-side is lower than for other spider mite species owing to the typical vivid pigmentation of this mite.

Thus, traits that compose the apparently striking appearance of *P. citri* seem highly functional from the viewpoint of antipredator defence. We hypothesise that lying down with protective setae represents an alternative antipredator defence for spider mites to producing protective webs. That is, *P. citri* does not have to produce protective webs that are costly in terms of reduced fecundity (Oku et al. 2009), and mites that produce protective webs do not have to lie down most of the time or possess long setae that might be a hindrance when living under a protective web.

*P. citri* disperses using air currents (Fleschner et al. 1956). However, its long dorsal setae are seemingly not essential for aerial dispersal because spider mites with short dorsal setae, such as *Tetranychus urticae* Koch and *T. kanzawai*, also disperse aerially (Smitley and Kennedy 1985; Osakabe et al. 2008). Although adult *P. citri* females with long dorsal setae appeared to be well defended against predatory mites, juvenile *P. citri* with shorter setae may have less protection against predators. How juvenile mites are defended against predators remains to be examined in future studies.

Since predatory mites sometimes attack *P. citri* from above (Additional file 2), *P. citri* has to defend in all directions except from below, where it is defended by the leaf surface. This must explain why the dorsal setae of *P. citri* are arranged and extend in all upward directions.

Although the constitutive antipredator defence of *P. citri* by lying down with protective setae is effective against predatory mites, it is totally ineffective against gigantic intraguild predators: lying *P. citri* individuals are often consumed together with leaves by swallowtail caterpillars (Shirotsuka and Yano 2012). Although *P. citri* and swallowtail caterpillars overlap on citrus host plants in time and space, the caterpillars are seemingly less harmful to *P. citri* than are predatory mites because the caterpillars forage randomly with respect to the presence of *P. citri* (Shirotsuka and Yano 2012), while predatory mites actively search for prey mites using various prey derived cues (e.g. Vet and Dicke 1992; Pratt and Croft 1999; Shinmen et al. 2010). From this viewpoint, the fact that *P. citri* does not move in response to external stimuli strongly suggests that the benefits of avoiding predatory mites are larger than the costs of coincidental intraguild predation that are relatively rare event for the mite.

## Electronic supplementary material

Additional file 1: **The moment of an unsuccessful attack (1).** Lying *P. citri* adult females never moved in response to the predatory mite *E. sojaensis*. Before long, the predator gave up and stopped approaching *P. citri* because of the interfering setae. (MPEG 3 MB)

Additional file 2: **The moment of an unsuccessful attack (2).** A female *E. sojaensis* that was attempting to approach the body surface of a lying *P. citri* female was repelled by an elastic seta (at the indicated time point of 16:25:34). (MPEG 2 MB)

## References

[CR1] Dawkins R, Krebs JR (1979). Arms races between and within species. Proc R Soc Lond B.

[CR2] Ehara S, Gotoh T (1992). Descriptions of two Panonychus spider mites from Japan, with a key to species of the genus in the world (Acari: Tetranychidae). Appl Entomol Zool.

[CR3] Fleschner CA, Badgley ME, Ricker DW, Hall JC (1956). Air drift of spider mites. J Econ Entomol.

[CR4] Fukaya M, Uesugi R, Ohashi H, Sakai Y, Sudo M, Kasai A, Kishimoto H, Osakabe M (2013). Tolerance to solar ultraviolet-B radiation in the citrus red mite, an upper surface user of host plant leaves. Photochem Photobiol.

[CR5] Inoue K, Osakabe M, Ashihara W (1987). Identification of pesticide resistant Phytoseiid mitepopulations in citrus orchards, and on grapevines in glasshouses and vinyl-houses (Acarina: Phytoseiidae). Jap J Appl Entomol Zool.

[CR6] Jones VP, Parrela MP (1984). Intratree regression sampling plans for the citrus red mite (Acari: Tetranychidae) on lemons in southern California. J Econ Entomol.

[CR7] Katayama H, Masui S, Tsuchiya M, Takara A, Doi M, Kaneko S, Saito T (2006). Density suppression of the citrus red mite Panonychus citri (Acari: Tetranychidae) due to the occurrence of Neoseiulus californicus (McGregor)(Acari: Phytoseiidae) on Satsuma mandarin. Appl Entomol Zool.

[CR8] McMurtry JA, Croft BA (1997). Life-styles of phytoseiid mites and their roles in biological control. Annu Rev Entomol.

[CR9] McMurtry JA, Huffaker CB, van de Vrie M (1970). Ecology of tetranychid mites and their natural enemies: A review. I. Tetranychid enemies: their biological characters and the impact of spray particles. Hilgardia.

[CR10] Mori K, Saito Y, Sakagami T (1999). Effects of nest web and female attendance on survival of young in the subsocial spider mite Schizotetranychus longus (Acari: Tetranychidae). Exp Appl Acarol.

[CR11] Nedelcu AM, Driscoll WW, Durand PM, Herron MD, Rashidi A (2011). On the paradigm of altruistic suicide in the unicellular world. Evolution.

[CR12] Nguyen TV, Shih C-IT (2012). Life-table parameters of Neoseiulus Womersleyi (Schicha) and Euseius Ovalis (Evans) (Acari: Phytoseiidae) feeding on six food sources. Int J Acarol.

[CR13] Oku K, Magalhaes S, Dicke M (2009). The presence of webbing affects the oviposition rate of two-spotted spider mites, Tetranychus urticae (Acari: Tetranychidae). Exp Appl Acarol.

[CR14] Osakabe M, Inoue K, Ashihara W (1986). Feeding, reproduction and development of Amblyseius sojaensis Ehara (Acarina: Phytoseiidae) on two species of spider mites and on tea pollen. Appl Entomol Zool.

[CR15] Osakabe M, Isobe H, Kasai A, Masuda R, Kubota S, Umeda M (2008). Aerodynamic advantages of upside down take-off for aerial dispersal in Tetranychus spider mites. Exp Appl Acarol.

[CR16] Pratt PD, Croft BA (1999). Expanded distribution of the bamboo spider mite, Schizotetranychus longus (Acari: Tetranychidae), and predation by Neoseiulus fallacies (Acari: Phytoseiidae). Acarologia.

[CR17] Sabelis MW, Bakker FM (1992). How predatory mites cope with the web of their tetranychid prey: a functional view on dorsal chaetotaxy in the Phytoseiidae. Exp Appl Acarol.

[CR18] Saito Y (1983). The concept of “life types” in Tetranychidae. An attempt to classify the spinning behaviour of Tetranichidae. Acarologia.

[CR19] Saito Y (1986). Biparental defence in a spider mite (Acari: Tetranychidae) infesting Sasa bamboo. Behav Ecol Sociobiol.

[CR20] Saito Y (1986). Prey kills predator: counter-attack success of a spider mite against its specific phytoseiid predator. Exp Appl Acarol.

[CR21] Shimoda T, Kishimoto H, Takabayashi J, Amano H, Dicke M (2009). Comparison of thread-cutting behavior in three specialist predatory mites to cope with complex webs of Tetranychus spider mites. Exp Appl Acarol.

[CR22] Shinmen T, Yano S, Osakabe M (2010). The predatory mite Neoseiulus womersleyi (Acari: Phytoseiidae) follows extracts of trails left by the two-spotted spider mite Tetranychus urticae (Acari: Tetranychidae). Exp Appl Acarol.

[CR23] Shirotsuka K, Yano S (2012). Coincidental intraguild predation by caterpillars on spider mites. Exp Appl Acarol.

[CR24] Smitley DR, Kennedy GG (1985). Photo-oriented aerial-dispersal behavior of Tetranychus urticae (Acari: Tetranychidae) enhances escape from the leaf surface. Ann Entomol Soc Am.

[CR25] Sudo M, Osakabe M (2011). Do plant mites commonly prefer the underside of leaves?. Exp Appl Acarol.

[CR26] Vet LEM, Dicke M (1992). Ecology of infochemical use by natural enemies in a tritrophic context. Annu Rev Entomol.

[CR27] Wanibuchi K, Saito Y (1983). The process of population increase and patterns of resource utilization of two spider mites, Oligonychus ununguis (Jacobi) and Panonychus citri (McGregor), under experimental conditions (Acari: Tetranychidae). Res Popul Ecol.

[CR28] Yano S (2012). Cooperative web sharing against predators promotes group living in spider mites. Behav Ecol Sociobiol.

